# Impact of a social media post on improving an existing residency program

**DOI:** 10.1093/ajhp/zxae344

**Published:** 2024-11-12

**Authors:** Kavish Choudhary, David Hager, Lynn Thoma, Erin R Fox

**Affiliations:** Department of Pharmacy, University of Utah Health, Salt Lake City, UT, and University of Utah College of Pharmacy, Salt Lake City, UT, USA; Visante, St. Paul, MN, USA; Visante, St. Paul, MN, and Purdue University College of Pharmacy, West Lafayette, IN, USA; Department of Pharmacy, University of Utah Health, Salt Lake City, UT, and University of Utah College of Pharmacy, Salt Lake City, UT, USA

**Keywords:** accreditation, preceptor, residency training, resiliency, social media

## Abstract

**Purpose:**

A social media post prompted reflection and changes to a longstanding pharmacy residency program. Pharmacy leadership utilized consultants with expertise in pharmacy residency training to help facilitate change and introduce new ideas to tenured staff. Other health systems working to continuously improve their residency programs may benefit from our example.

**Summary:**

The guiding principles of the changes were to align with the new harmonized residency program standards and reduce preceptor burden and potential burnout. Changes were made to the entire program’s design, including new leadership, changes to committee structure, reduced requirements for preceptors that were not required in the standards, and resident recruitment and onboarding.

**Conclusion:**

We encourage residency programs to regularly evaluate program structure, design, and conduct to ensure alignment with the organization’s evolving priorities as well as its mission and vision.

University of Utah Health’s residency program was established in 1991 and received the American Society of Health-System Pharmacists (ASHP) Pharmacy Residency Excellence Award in 2011. This success led to the program’s expansion and growth from 5 to 6 residents per year to 22 to 25 per year. Residency training is a foundational aspect of the organization, which is a medium-sized health system with an academic medical center that is the primary medical training site for the state of Utah. The staff involved with the residency program are highly committed and invest significant time, often outside of regular work hours, into teaching and precepting. Yet, in January 2021 a negative social media post prompted reflection and change.

“Be aware of a program in Utah.” Those words appeared on Reddit, a social media website in which users can anonymously share content, such as text-based posts, photos, videos, and links in a bulletin board format. The poster, stating they were a staff member, shared residency program feedback warning prospective residents about a toxic culture of bullying, unrealistic expectations, and a track record of firing residents.^1^ As staff became aware of the post, leadership solicited feedback from the residency advisory committee, preceptors, and current and past residents. A unique characteristic of the university’s program is the longevity of the staff, including the residency leadership team and preceptors, most of whom are “homegrown” and have never worked or practiced in another health system. At the time of this social media post, staff were experiencing high levels of burnout, with only 27% stating “burnout is not a problem for me.” The social media post drew attention to the burnout leadership and staff members were experiencing, possibly causing them to take out their frustrations on the residents.

Pharmacy leadership began to look into ways to change the residency training program to address the concerns voiced in the post. However, while growing from within is a positive from a workforce and hiring perspective, the leaders had concerns about an “echo chamber” mentality and whether real change could happen solely from inside the pharmacy department. Therefore, they decided to obtain outside help from pharmacy consultants to overcome potential cultural barriers and time constraints. The leaders sought consultants with expertise in pharmacy residency training to conduct a complete review of the current state of the program and to provide recommendations and help implement change. This article provides an overview of the experience and process of making these needed changes, and it may benefit other health systems that are looking to continuously improve their residency programs.

## Making data-driven changes

The landscape of pharmacy education and practice has changed dramatically. For instance, the North American Pharmacist Licensure Examination pass rates declined from 97% in 2012 to 80% in 2022.^2^ Pharmacy school applications have also declined, with the average number of applicants per school decreasing 60% from 2015 to 2022.^3^ These data, combined with waning student interest in traditional residency training, reveal an evolving challenge of maintaining high standards.^3^ This shift grants students more leverage and intensifies the competition among residency programs to attract top candidates. Additionally, generational differences, a focus on work-life balance, and changing work priorities post pandemic created a need for a new kind of learning environment for residents.^2^ Consequently, practice advancement initiatives for pharmacists and layered learning models make attracting high-quality residents a professional priority. In 2023, ASHP provided the “Resource Guide for Well-Being and Resilience in Residency Training” to provide programs with needed tools to make program changes.^4^

The release of new ASHP harmonized accreditation standards^5^ and well-being resources^4^ provided the pharmacy leadership team and consultants with the perfect impetus to create a plan for the redesign of the residency program. Postgraduate year 1 (PGY1) residencies were divided into acute care or ambulatory care programs, with a separate program director for each. All staffing requirements were also made consistent among the various programs.

One key goal of the redesign targeted the culture of bullying the Reddit post described. The team’s philosophy in planning changes was to reduce workload, restore joy and job satisfaction for preceptors, and demonstrate a strong commitment to long-term staff. The redesign plan focused on 3 key areas: resources, expectations, and alignment.

Since the Reddit post, we have had a more thoughtful approach to our residency programs and overall structure. Previously, we missed signs of preceptor burnout; pharmacy residents make up about 2% of the full-time equivalents of our department but were getting a disproportionate share of resources and were often prioritized over preceptors and other staff. This may have created staff resentment and contributed to burnout. We acknowledged that we had opportunities to create a healthier workplace by prioritizing our preceptors and ensuring a positive learning environment for our residents.^6^

### Resources

Table 1 provides a summary of the changes made to reduce the resource burden of the residency program. Over the years, the number of preceptors involved in interviewing residency candidates grew to include over 70 staff members. Staff were burdened by covering for colleagues who were taken offline from patient care duties to interview residents. Through changing the interviewing approach, the number of staff involved in interviews was decreased by half, reducing the burden of cross covering and labor costs. Resident presentations and teaching requirements also caused significant burden without providing meaningful value to residents or the organization. For example, PGY1 residents were required to provide a clinical seminar in the early months of their residency. This seminar was not related to the resident’s rotations or longitudinal project or the program’s competency areas, goals, and objectives (CAGOs). These clinical seminars required hours of mentorship from preceptors outside of rotation teaching; therefore, the requirement was removed. Unnecessary evaluations via the PharmAcademic (McCreadie Group, Ann Arbor, MI) web-based platform (eg, midpoint evaluations for every rotation) were also discontinued to allow preceptors to spend more time with residents and less time documenting evaluations. University of Utah Health had developed a homegrown teaching certificate program that also required significant preceptor time. The team decided to outsource this program to reduce preceptor burden, offering residents the program provided through ASHP.^7^ Outsourcing the teaching certificate also allowed the elimination of a monthly teaching certificate committee meeting. Additional committee changes are described in Table 1.

**Table 1.** Changes to Residency Program

Area	Previous	Changes	Comments
PGY1 program	One program for 10 residents, including 2 first-year HSPAL residents	Single program split into 2 programs: Acute Care/PGY1 HSPAL and Ambulatory	Aligns with new ASHP guidance
PGY1 leadership	1 RPD and 2 RPC’s for 10 residents	3 RPDs (Acute, Ambulatory, and HSPAL) and 4 RPCs for 8 residents	Same RPD and RPC for both years of the 2-year HSPAL program
Presentation requirements	Required all PGY1 residents to present a clinical seminar, ACPE-accredited continuing education, abstract at national meeting, and platform presentation at regional residency conference	Clinical seminar requirement removed	RPDs continue to review presentation requirements
Teaching certificate	Homegrown program with committee and individual teaching mentors	Teaching certificate outsourced to ASHP program	Eliminates preceptor burden
Preceptor development	All preceptors were required to attend or obtain 5 hours of preceptor development annually; options were determined by a central committee without customization	Sub-RACs are tasked with identifying preceptor development customized to their program using the available resources of the organization	Allows RPDs to customize development for their preceptors
Residency advisory committee	Single overall RAC with subcommittees including Recruitment, Preceptor Expectations, Research and Scholarship, Preceptor Development and Teaching Certificate	Single overall RAC for organization-wide decisions with a sub-RAC for each program; some subcommittees elimated (Recruitment, Preceptor Expectations, Preceptor Development and Teaching Certificate)	Provides more autonomy to RPDs to customize their programs and involve preceptors in decision-making; allows faster changes from residents and frontline preceptors
Resident feedback to the program	Residents provided feedback in an after-hours meeting with RPDs; details of the feedback were not shared with RPDs, residents, or pharmacy leadership	Resident feedback provided in transparent grid noting action or comments; online form is available for any staff member to provide feedback	Goal is more transparency with all involved in residency training
Resident recruitment	Rubrics were not contemporary to changing applicant pool and were created prior to ASHP focus on diversity; U of U Health employees applying to residency were not handled based on employment best practices; preceptor time away from clinical practice for interviews was a staffing model burden	Rubrics were streamlined to improve application review efficiency, and accuracy and included updated organizational priorities; U of U Health employees are now interviewed when they meet organizational policy criteria; reduced number and hours preceptors engage in interview process	Standardized around virtual interview approach
Residency manual	Manual had expanded over many years to include all guidance a resident or preceptor might need, resulting in a 57+ page document; manual included elements specific to PGY1 program and all residents, creating confusion about what requirements applied to whom	Manual was streamlined to how U of U meets current ASHP accreditation requirements for elements germane to all programs; all other program-specific content is in appendices, reducing common manual to 30 pages and improving clarity	Goal is to provide centralized support for programs where it creates efficiency
Resident onboarding	Centralized resident onboarding had expanded the volume of content over the years based on preceptor and resident feedback; customization based on the incoming residents’ program and prior experience level was minimal	Core elements of successful onboarding were centralized and standardized for 2 cohorts of onboarding residents (PGY1 and newly hired PGY2 residents); PGY2 residents who completed PGY1 at U of U Health have customized onboarding schedules	RPD autonomy to customize the onboarding experience for residents ensures residents get the right information when they need it, not everything they could need all at one time
Project proposals and approval process	Project training was scholarship focused despite new organizational focus on QI for PGY1 resident projects, creating a mismatch; there was lack of transparency and clarity on the project approval process; approved projects did not always align with the organization’s goals and priorities	Resident training was revamped with QI focus; project sub-RAC was empowered to create a consistent project approval process that included stakeholders from typical bottlenecks; leadership review was added for alignment to organizational needs	Aligning resident projects with organizational and department strategic plans/yearly goals is essential to keeping efforts aligned and reducing bottlenecks (analytics, informatics, financial resources, etc)

Abbreviations: ACPE, Accreditation Council for Pharmacy Education; ASHP, American Society of Health-System Pharmacists; HSPAL, health-system pharmacy administration and leadership; PGY1, postgraduate year 1; QI, quality improvement; RAC, residency advisory committee; RPC, residency program coordinator; RPD, residency program director; U of U, University of Utah.

### Expectations

Some preceptors expected residents to clinically perform at the level of a seasoned practitioner at the start of residency, setting unrealistic expectations for the learners. These unrealistic expectations did not align with the ASHP CAGOs. Unrealistic expectations also set up residents to “fail” in subsequent rotations; thus, the next preceptor would receive the information that the resident “wasn’t meeting expectations.” The consultants identified an education gap within our internal preceptor group regarding the ASHP residency requirements and the lack of a standard definition for resident evaluations. The consultants worked with internal preceptors to realign expectations and educate preceptors about the educational philosophy that underpins the residency learning system and how to apply a standard definition for resident evaluations, challenging the more academic-focused “pass or fail” approach of the past.

### Alignment

Resident projects did not always align with departmental priorities or organizational goals. A key change was to involve the pharmacy leadership team in approving the residency project ideas. Also, instead of completing a research education program targeted towards prospective study designs or bench research, our PGY1 residents completed formal education on conducting quality improvement projects through the internal resources of our organization. The leadership team also changed the structure of residency manuals, the preceptor handbook, and policies and procedures to align with the new harmonized standards and ensure clarity for resident and preceptor expectations. For many years, the process had been to update the prior year’s manual. With the release of the new ASHP harmonized accreditation standards, it was recognized that a back-to-basics approach was needed, which required adding or revising these documents only when it was truly necessary. This in turn reduced the administrative burden on residents and preceptor, resulted in a considerably shorter residency manual.

### Initial experience and next steps

The primary reason for the residency program redesign was to create a workplace where people are proud of their profession and want to encourage and assist younger pharmacists in pursuing successful careers. The team believes in growing and supporting a new generation of residency program leadership, which requires finding an optimal balance between program-level control and overarching support.

The new residency structure allows for the assignment of a smaller number of residents to each program director. This ensures more support for each resident and less administrative burden for the directors. While this number varies based on program demand and individual program director capacity, residency program directors were encouraged to recruit for the number of residents they believed the preceptors in their area could support using published literature on the number of learners and its relationship to pharmacist burnout.^8^ Additionally, the new program directors identified new learning experience sites that offered more options to the residents in terms of both preceptors and learning experiences. Succession planning and more formal leadership opportunities for preceptors are also important elements in the new structure.

Prior to redesign efforts, virtually all decision-making for all programs was controlled by a single residency advisory committee (RAC). Each program now has its own RAC, and efforts are underway to restructure the decisions and activities an overall RAC should control. This redesign is a continuous process improvement activity, with the goal being to support program directors and gain efficiency with central activities while at the same time allowing each program more autonomy to make decisions germane to their residency program.

## Discussion

Pharmacy leadership and residency program directors should be proactive in routinely reevaluating their residency programs and improving their residency recruitment strategies in the face of a continuous decline in supply. They should ensure their program structure, design, and conduct are aligned with their organization’s changing priorities as well as its mission and vision, and maintain the program’s integrity and rigor while supporting residents and preceptors in new ways. This includes challenging the notion that traditional methods of pharmacy training are the best and only options. It is also important to determine where program centralization provides value and when program autonomy enhances efficiency and customization to meet resident needs. Program directors should focus on the fundamental requirements outlined in the CAGOs and the harmonized residency accreditation standards, rather than relying on institutional memory.

The residency program’s redesign at University of Utah Health will never be fully complete but has received positive feedback, through discussions at RAC and department meetings, from preceptors who feel less burdened and more excited to teach. These positive results link to our goal of bringing back the joy of precepting and teaching. A key next step is to create balance and equity between the various programs. There are still perceptions that one program may be less intense than another regarding staffing, rotations, call, projects, presentations, and overall expectations. Another goal is to create a true feedback mechanism for the residency program leadership and residents. The team is committed to doing more than just gathering feedback; they are also committed to transparency. This means showing how the feedback was acted upon, and if it was not, providing the reason why.

## Conclusion

A social media post prompted the redesign of a longstanding pharmacy residency program. Changes were made to reduce the resource burden associated with the program, decrease burnout among preceptors and program directors, address unrealistic expectations of residents, align resident projects with organizational priorities and goals, and provide residents with more support.

## Data Availability

The data underlying this article are available in the article.

## References

[1] Reddit. Be aware of a program in Utah. Accessed May 23, 2024. https://www.reddit.com/r/PharmacyResidency/comments/kuv7d6/be_aware_of_a_program_in_utah/

[2] Brandon HH , Romanelli F. The North American Pharmacist Licensure Examination (NAPLEX) pass rate conundrum. Am J Pharm Educ. 2024;88(5):100701. 38641172 10.1016/j.ajpe.2024.100701

[3] National Matching Services Inc. ASHP match statistics. Accessed May 25, 2024. https://natmatch.com/ashprmp/stats.html

[4] American Society of Health-System Pharmacists. ASHP resource guide for well-being and resilience in residency training. Accessed August 10, 2024. https://www.ashp.org/-/media/assets/professional-development/residencies/docs/ASHP-Well-Being-Resilience-Residency-Resource-Guide-2023.pdf

[5] American Society of Health-System Pharmacists. ASHP accreditation standards for postgraduate residency programs. Accessed May 25, 2024. https://www.ashp.org/-/media/assets/professional-development/residencies/docs/examples/ASHP-Accreditation-Standard-for-Postgraduate-Residency-Programs.pdf

[6] Hardeman S , Musselman M, Weightman S, Gosser R, Derry K, MacDonald E. A call to action: how pharmacy leadership can manage burnout and resilience. Am J Health-Syst Pharm. Published online July 29, 2024. doi: https://doi.org/10.1093/ajhp/zxae219 39073434

[7] American Society of Health-System Pharmacists. Teaching Certificate for Pharmacists. Accessed August 26, 2024. https://elearning.ashp.org/products/11585/teaching-certificate-for-pharmacists

[8] May A , Raber H, Tingey B, et al The association between number of learners and pharmacist and technician levels of burnout. Am J Health-Syst Pharm. 2024;81(10):370-384. 38237931 10.1093/ajhp/zxad339

